# Genetically engineered M13 phage-mediated H9N2 DNA vaccine with enhanced mucosal and systemic immune responses in mice

**DOI:** 10.1080/10717544.2026.2629037

**Published:** 2026-02-11

**Authors:** Xiaohua Wang, Zhi Zhao, Mingze Shi, Shangen Xu, Xin Zhou, Kai Zhao

**Affiliations:** a Zhejiang Key Laboratory for Restoration of Damaged Coastal Ecosystems, Zhejiang International Science and Technology Cooperation Base for Biomass Resources Development and Utilization, Taizhou Key Laboratory of Biomedicine and Advanced Dosage Forms, School of Life Sciences, Taizhou University, Zhejiang, Taizhou, People's Republic of China; b College of Veterinary Medicine, Institute of Comparative Medicine, Yangzhou University, Yangzhou, Jiangsu, People's Republic of China

**Keywords:** H9N2 avian influenza virus, nanovaccine, M13 phage, quaternized chitosan nanoparticle, gene delivery vehicle

## Abstract

Vaccine adjuvants and delivery systems have long been used in DNA vaccines to enhance immunogenicity. In this study, we developed a DNA vaccine delivery platform by combining *N*-2-hydroxypropyl trimethyl ammonium chloride chitosan/carboxymethyl chitosan nanoparticles (N-2-HACC/CMCS NPs) with a genetically engineered M13 phage containing the HA gene of H9N2 AIV (HA-M13). The composite NPs (HA-M13/N-2-HACC/CMCS) had an average particle size of 135.24 ± 4.36 nm, and the HA gene encapsulated in the composite NPs could be expressed *in vitro*. Additionally, the N-2-HACC/CMCS NPs exhibited high stability and effectively protected the HA gene and M13 phage from degradation while sustaining antigen release. Furthermore, the N-2-HACC/CMCS NPs promoted the maturation of DC2.4, enhanced MHC I and MHC II pathways and improved cellular, humoral, and mucosal immune responses. Mice immunized with HA-M13/N-2-HACC/CMCS via nasal and intramuscular injections presented higher anti-H9N2 AIV antibody titers than those given the commercial vaccine. Lymphocyte proliferation, as well as the levels of the cytokines IL-2, IL-4, IFN-*γ*, CD4^+^, and CD8^+^ T lymphocyte levels, also significantly increased. The nanovaccine provided effective protection against H9N2 AIV infection for 154 days postimmunization, surpassing the 120-day protection provided by the commercial vaccine. Consequently, the N-2-HACC/CMCS NPs loaded with M13 phages exhibit significant potential as vaccine adjuvants and mucosal immune delivery system.

## Graphical abstract

**Figure:**
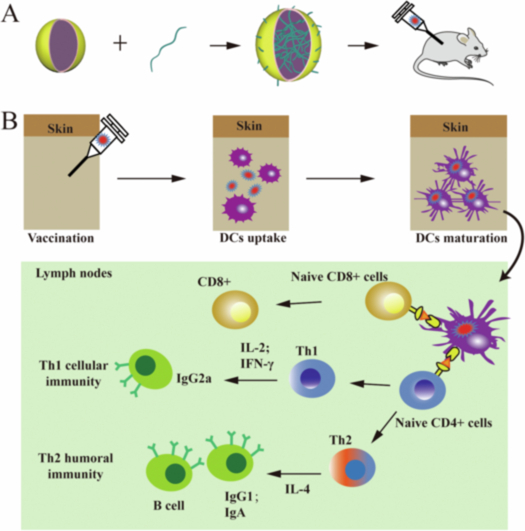

## 1. Introduction

Avian influenza (AI) is an acute infectious disease caused by a subtype of influenza A virus, commonly referred to as the European chicken plague (Fellahi et al. 2021). Among them, H9N2 avian influenza virus (AIV) is a low-pathogenic AIV subtype with zoonotic potential (Song and Qin 2020; Um et al. 2021). Existing commercial H9N2 AI vaccines predominantly consist of inactivated formulations. Although these vaccines stimulate humoral immunity, they have limitations like long production times, and inability to induce cellular and mucosal immune responses against pandemic strains (Gu et al. 2017; Wang et al. 2019; Su et al. 2020; Fellahi et al. 2021). While recombinant and subunit vaccines are capable of eliciting humoral, cellular, and mucosal immune responses, they often fail to confer long-term immune protection (Song et al. 2020; Yin et al. 2020; Song et al. 2021). Given the constant mutation of AIVs, novel vaccines supported by new technologies are emerging. DNA vaccine platforms permit real-time sequence modifications to match viral mutations, providing unparalleled responsiveness to virus evolution (Lu et al. 2024). In addition, DNA vaccines show promise due to their broad antigen selection, short development cycle, and ability to stimulate both humoral and cellular immunity (Richner et al. 2017; Meyer et al. 2018; Fauci et al. 2021). Therefore, there is an urgent need to develop AIV DNA vaccines that exhibit high immunogenicity, long-lasting immune effects, and convenient storage and transportation conditions. However, the use of DNA is hampered by several enzymes and therefore need for development of novel delivery systems for DNA vaccines.

Nanomaterials provide excellent stability, extend drug release, and enhance the uptake rate by antigen-presenting cells (APCs), thereby augmenting immunogenicity (Liu et al. 2023). A variety of nanomaterials, including chitosan nanoparticles (NPs), gold NPs, carbon nanotubes, mesoporous silica NPs, calcium phosphate NPs, aluminum NPs, liposomes, and polylactic acid, can be employed as immune adjuvants and delivery systems (Levingstone et al. 2020; Li et al. 2020; Mishra et al. 2021; Nirwan et al. 2021). When utilized as delivery carriers, NPs facilitate the transport of loaded antigens to primary APCs, thereby fostering immune responses (Shen et al. 2018). Vaccine encapsulation within nanoparticle systems has been extensively researched due to their convenience, rapidity, ability to induce mucosal and systemic antibody immune responses (Wan et al. 2025). However, some nanovaccine delivery systems still face challenges related to sophisticated manufacturing processes for large-scale production, relatively high costs, and limited biodegradability. In contrast, chitosan-based NPs, including chitosan derivatives, are derived from abundant natural polysaccharides and can be fabricated using simple and mild processes with favorable biodegradability. Our group synthesizes water-soluble chitosan derivatives: N-2-hydroxypropyl trimethyl ammonium chloride chitosan (N-2-HACC) and carboxymethyl chitosan (CMCS) that remain chitosan desirable properties (Jin et al. 2013; Gao et al. 2022). Moreover, the negatively charged CMCS and positively charged N-2-HACC were combined to create N-2-HACC/CMCS NPs through cross-linking.

M13 phages exhibit inherent pathogenic properties and serve as agonists for the Toll-like receptor (TLR) pathway (Dong et al. 2022). When intergrated with chitosan and its derivatives in phage delivery systems, these phages are internalized by cells through endocytosis and subsequently processed by APCs (Bartolacci et al. 2018; Gomes-Neto et al. 2018). Subsequently, antigens are processed and presented through the major histocompatibility complex (MHC) class I and class II pathways, thereby eliciting both cellular and humoral immune responses (Kim et al. 2012; Aghebati-Maleki et al. 2016). This process generates specific CD4^+^ and CD8^+^ T lymphocyte responses. The integration of chitosan and its derivatives into phage-based systems also promotes sustained release, extending the protection offered by the prepared vaccines (Abdelsattar et al. 2019; Gondil et al. 2020).

In this study, we developed a DNA vaccine delivery platform by combining chitosan quaternary ammonium salt NPs with bacteriophages. This work aims to enhance the stability of bacteriophages, improve antigen delivery efficacy, and prolong the release time of the vaccines to enhance immunogenicity. We further evaluated the properties of NPs and their ability to activate DC2.4 cells. Finally, we validated the protective effect through *in vivo* animal experiments, assessing the clinical potential of immune prophylaxis against the spread of H9N2 AIV.

## 2. Materials and methods

### 2.1. Materials

Chitosan with a molecular weight of 71.3 kDa and a deacetylation degree of 80%–95% was purchased from Sinopharm Chemical Reagent Co., Ltd. (Shanghai, China). H9N2 AIV HA antibody was purchased from Vivo Biotechnology Co., Ltd. (Nanjing, China) and goat anti-mouse IgG-HRP were purchased from Sangon Biotech (Shanghai, China). The DL2000 Plus DNA marker and 180 kDa prestained protein marker were purchased from Novizan Co., Ltd. (Nanjing, China). The commercial combined inactivated vaccine against Newcastle disease (ND) and AI was purchased from Harbin Pharmaceutical Group Co., Ltd. (Harbin, China). A DNA extraction kit was purchased from Corning Life Sciences Co., Ltd. (Wujiang, China), and an enzyme-linked immunosorbent assay (ELISA) was purchased from Shanghai Enzyme-linked Biotechnology Co., Ltd. (Shanghai, China). PE-conjugated CD86 monoclonal antibody, PE-conjugated CD80 monoclonal antibody, PE-conjugated CD4 monoclonal antibody, APC-conjugated CD8 monoclonal antibody and FITC-conjugated CD3 monoclonal antibody were purchased from Thermo Fisher Scientific Co., Ltd. (Shanghai, China). H9N2 AIV (A/Chickens/XZ-3/2019), M13 phage with eukaryotic promoter CMV (a titer of 10^12^ pFU/mL) and *E. coli* (ER2738) were obtained from the School of Veterinary Medicine, Yangzhou University. Unless otherwise stated, all other reagents were purchased from the Nanjing Well Offer Biotechnology Co., Ltd. (Nanjing, China).

### 2.2. Cell lines

DC2.4 (mouse bone marrow-derived dendritic cell) was provided from the Institute of Comparative Medicine in Yangzhou University. DC2.4 were cultured in Dulbecco's modified Eagle's medium (DMEM) medium (Gibco, NY, USA) supplemented with 10% bovine serum (FBS, Clark, VA, USA) and 1% antibiotics (Gibco, NY, USA).

### 2.3. Construction of M13 phage with HA gene of H9N2 AIV

To construct HA-M13, the HA gene of H9N2 AIV was inserted into a plasmid containing a eukaryotic expression cassette, and the complete expression cassette was then cloned into the M13 phage genome. According to the manufacturer's instructions, the RNA of H9N2 AIV and DNA of M13 phage were extracted using FastPure Viral DNA/RNA Mini Kit, respectively. H9N2 cDNA was obtained by reverse transcription of RNA using HiScript III All in one RT SuperMix. The HA gene was amplified by polymerase chain reaction (PCR) on the H9N2 cDNA template (Table S1). The HA gene was then inserted into the plasmid PUC57, which carried the eukaryotic expression cassette for the HA gene expression cassette. The linearized M13 genome was obtained by PCR on the M13 phage DNA template (Table S1). The HA gene expression cassette and linearized phage DNA were recombined into a loop using the ClonExpress Ultra One Step Cloning Kit V2, and the resulting product was transformed into TG1-responsive cells. After overnight cultivation at 37 °C, bacteriophage plaques were selected for identification and sequencing to obtain HA-M13.

To obtain a large number of purified phages, HA-M13 with the correct sequence was cultured overnight in *Escherichia coli* ER2738 according to the previous reports (Singh et al. 2018). Briefly, after overnight, the supernatant of culture medium was centrifuged and mixed with 5% NaCl/PEG-8000 solution. Then standing overnight at 4 °C, after which the precipitates were collected and resuspended in PBS. Finally, PEG-8000 was removed through a 30 kDa dialysis membrane to obtain purified HA-M13. The morphology and Zeta potential of the HA-M13 examined by transmission electron microscopy (TEM, Royal Philips, Netherlands) and 90plus PALS (Brookhaven Instruments, USA).

### 2.4. Preparation and characterization of the N-2-HACC/CMCS NPs loaded with HA-M13

The N-2-HACC was synthesized as described in a previous study (Jin et al. 2013). The HA-M13/N-2-HACC/CMCS preparation process is outlined as follows. Initially, 4 mL of the 1 mg/mL N-2-HACC solution was added to a beaker at 25 °C. Subsequently, 2 mL of the 10^8^ pFU/mL HA-M13 was incorporated, followed by magnetic stirring at 300 r/min for 10 min. The stirring speed was then increased to 1100 r/min and maintained for additional 5 min. In the second step, 1 mL of the 1.5 mg/mL CMCS solution was added to the resulting mixture, which was stirred at 1100 r/min for 40 min. Eventually, the supernatant was separated by centrifugation at 4 °C and 12,000 r/min for 30 min. The pellets obtained were subsequently resuspended in sterile ddH_2_O and freeze-dried under vacuum conditions to generate the N-2-HACC/CMCS NPs loaded with HA-M13 (HA-M13/N-2-HACC/CMCS).

The morphological and surface characteristics of the HA-M13/N-2-HACC/CMCS were examined utilizing HT7800 TEM (Royal Philips, Netherlands). The particle size and zeta potentials were measured using a Nano ZS90 from Malvern Instruments (Malvern, Worcestershire, UK). The encapsulation efficiency (EE) and loading capacity (LC) were quantified *via* the following formula, which was replicated at least three times; where W_0_ represents the total amount of M13 phage added, W_1_ denotes the amount of free M13 phage, and W refers to the weight of the HA-M13/N-2-HACC/CMCS.

(1) $$\mathrm{EE}(\%)=(W_{0}-W_{1})/W_{0}\times 100\%$$

(2) $$\mathrm{LC}(\%)=(W_{0}-W_{1})/W\times 100\%$$

### 2.5. *In vitro* expression and release assay of the HA-M13/N-2-HACC/CMCS

DC2.4 cells were seeded into a 96-well plate containing DMEM supplemented with 10% FBS and 1% penicillin–streptomycin and were cultured overnight. A total of 5 × 10^5^ cells per well were seeded, cultured for 24 h, and then exposed to H9N2 AIV, M13, and HA-M13/N-2-HACC/CMCS for 6‒8 h. Cells were collected for Western blot analysis, and the H9N2 AIV HA protein was used as a positive control for the HA-M13/N-2-HACC/CMCS protein assay. Briefly, samples were incubated with primary antibody (mouse antibody against AIV H9N2 HA) for 3 h, followed by incubation with secondary goat anti-mouse IgG-HRP for 3 h, after which they were incubated with Western blotting substrate (Beyotime, China), after which images were captured using an enhanced chemiluminescence (ECL) system (Beyotime, China).

For *in vitro* release, 0.01 g of HA-M13/N-2-HACC/CMCS was dissolved in 1 mL of phosphate-buffered saline (PBS) buffer (pH 7.2) and fully stirred at 100 r/min at 4 °C. Samples were withdrawn at predetermined time points (0, 3, 6, 9, 12, 24, 36, 48, 60, 72, 96, 120, 144, and 168 h). We conducted each experiment three times and generated the *in vitro* cumulative release curve of HA-M13/N-2-HACC/CMCS by plotting the mean value of the cumulative release rate of HA-M13 versus time.

### 2.6. Stability assay of the HA-M13/N-2-HACC/CMCS

The adjuvant dissolution method employed showed not significant impact on the response. To prepare the samples, 0.1 g of the HA-M13/N-2-HACC/CMCS was added to 50 mL of PBS solution at pH 5.6, 7.0, and 10.0, followed by incubated at 20–25 °C for 7, 14, and 30 days, respectively. The HA-M13/N-2-HACC/CMCS samples were stored at 25 °C and refrigerated at 4 °C and −20 °C for 7, 14, and 30 days. Subsequently, all three samples were transferred to a temperature-controlled stability incubator set at 37 °C for the duration. The particle size and morphology were observed using a TEM, and stability was assessed by measuring the transmittance at 500 nm using a spectrophotometer.

### 2.7. The safety assay of the HA-M13/N-2-HACC/CMCS

*In vitro* cytotoxicity was evaluated using a cell counting kit-8 (CCK-8) assay in DC2.4 cells. A total of 1 × 10^5^ DC2.4 cells per well were seeded in a 96-well plate containing DMEM supplemented with 10% heat-inactivated FBS and 1% penicillin-streptomycin. After overnight culture, the HA-M13, N-2-HACC/CMCS, or HA-M13/N-2-HACC/CMCS with different concentrations, were added into wells and incubated for 24 h. Subsequently, the cells were treated with a CCK-8 solution, and the absorbance was measured with a wavelength of 450 nm. The results were expressed as means ± SD (*n* = 3).

For *in vivo* safety evaluation, thirty SPF BALB/c male mice aged 6–8 weeks, were randomly assigned to six groups based on a predetermined randomization list. Mice in groups 1 through 6 received intramuscular injections of 100 μL of normal saline, HA-M13 (10^8^ pFu/mL), N-2-HACC/CMCS NPs (500 μg/mL), HA-M13/N-2-HACC/CMCS (50 μg/mL), HA-M13/N-2-HACC/CMCS (100 μg/mL), and HA-M13/N-2-HACC/CMCS (500 μg/mL), respectively. Mouse weights were measured on days 7, 14, and 21 after administration. Additionally, the influence on the morphology of the lung, spleen, kidney and liver of mice was test by hematoxylin and eosin (H&E) staining.

### 2.8. Induction of DC2.4 maturation and detection of cellular uptake efficiency

For cell uptake analysis, FITC-N-2-HACC was synthesized according to our previously published methods (Gao et al. 2022), then replace N-2-HACC with FITC-N-2-HACC to get FITC-HA-M13/N-2-HACC/CMCS. The DC2.4 cells were cultured in confocal dish, and 100 μL of FITC-HA-M13/N-2-HACC/CMCS was added to the co-culture. At various time points (1, 2, 4, 8, 12, 18, and 24 h), the medium was discarded, and the cells were fixed with 2 mL of 4% paraformaldehyde. Afterward, the cells were washed four times with PBS, stained with DAPI for nuclear staining, and mounted with an anti-fluorescence quenching mounting solution. Finally, the cells were visualized under a laser confocal microscope.

### 2.9. Animal immunization and challenge

The 60 male BALB/c mice were randomly divided into six groups with 10 mice in each group. Mice in Group 1 were immunized intramuscularly (i.m.) with PBS buffer, mice in Group 2 were immunized i.m. with the blank N-2-HACC/CMCS NPs, mice in Group 3 were immunized i.m. with the HA-M13, mice in Group 4 were immunized i.m. with the commercially combined inactivated vaccine against ND and AI, mice in Group 5 were immunized i.m. with the HA-M13/N-2-HACC/CMCS, mice in Group 6 were immunized intranasally (i.n.) with the HA-M13/N-2-HACC/CMCS. The vaccinations were administered at a dose of 300 μL on days 0 and 14, respectively. On day 21, six mice were randomly selected from each group and infected intramuscularly with 100 μL of the standard virulent H9N2 AIV at a viral titer of 10^7.0^ TCID_50_. Furthermore, on day 3 after the challenge, the lungs of three mice from each group were collected to determine the viral titer. The weight of mice which challenged with H9N2 AIV was continuously monitored and recorded for 21 days following the challenge. Mice were euthanized if their body weight loss reached 20% of their initial body weight. Subsequently, the infected mice and corresponding negative control mice (from unchallenged mice in group 1) were euthanized, and their lungs, hearts, and kidneys were collected for histological examination by staining. The timeline of immunization and challenge is presented in Figure S1.

### 2.10. Serological examination by ELISA

Serum samples were collected from the tail vein of mice 1 day prior to the initial vaccination, and at 7, 14, 21, 28, 35, and 42 days postvaccination (*n* = 5). The concentrations of IgG, IgG1, IgG2a, IL-2, IFN-*γ*, and IL-4 in serum, and sIgA levels in the feces were determined using ELISA, according to the manufacturer's instructions. All assays were independently replicated three times.

### 2.11. Hemagglutination inhibition assay

The hemagglutination inhibition (HI) assay was performed according to previously published methods (Gilchuk et al. 2019). Briefly, 25 μL (4 hemagglutination units) standard virulent H9N2 AIV were incubated with 25 μL two-fold serial dilutions of serum samples for 1 h at room temperature. Then, the serum-virus mixture was incubated with 50 μL of 0.5% (vol/vol) cock red blood cells for 30 min at room temperature. The HI titer was defined as the lowest serum dilution that inhibited hemagglutination of red blood cells.

### 2.12. Evaluation of lymphocyte subpopulations by flow cytometry assay

To evaluate the influence of HA-M13/N-2-HACC/CMCS on cell-mediated immune responses, we performed a flow cytometry assay to assess lymphocyte activation and T lymphocyte response in the spleen. Splenocytes from immunized mice were harvested 24 days after the first vaccination. Under aseptic conditions, the mice were dissected, and the spleen was meticulously removed and placed in a dish containing RPMI-1640 medium. The spleen was then homogenized and centrifuged at 2000 r/min for 8 min, and the supernatant was discarded. To eliminate red blood cells, three to five times the volume of erythrocyte lysis solution was added, and the mixture was incubated for 1–2 min, followed by centrifugation at 400–500 r/min for 5 min. The sediment was collected and washed with PBS buffer (pH 7.2). The resulting splenocytes were resuspended in 1  mL of serum-free RPMI-1640 medium and observed and counted under a microscope.

Additionally, mouse spleens were collected 21 days after the first immunization for T-cell subtype (CD3, CD4, and CD8) analysis using flow cytometry, as previously described (Bi et al. 2020; Pishesha et al. 2022). Splenocyte proliferation experiments were performed on CFSE-stained splenocytes. Spleen lymphocytes (2 × 10^6^ cells/mL) were incubated with FITC-conjugated CD3, PE-conjugated CD4, and APC-conjugated CD8 monoclonal antibodies (Carlsbad, CA, USA) at 4 °C for 30 min (Ou et al. 2013; Liu et al. 2020). A CytoFLEX flow cytometer (Beckman Coulter, Shanghai, China) was employed to determine lymphocyte subpopulations and proliferation. Data analysis was performed using FlowJo software.

### 2.13. Statistical analysis

All the results were expressed as mean values ± standard deviation (SD). One-factor analysis of variance (ANOVA) was employed to assess the statistical differences among different groups with GraphPad Prism 8 software. The significant differences between groups with *P* values of <0.05 and <0.01 were considered to be statistically significant.

## 3. Results

### 3.1. Construction of the HA-M13

**Figure 1.**
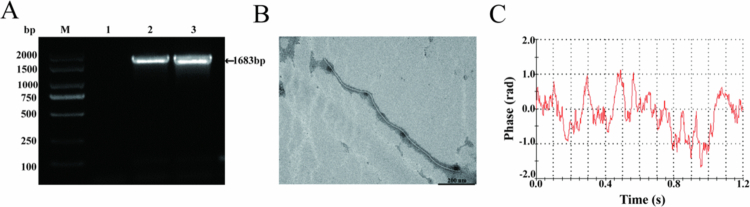
Physicochemical characterization of the HA-M13. (A) PCR amplification products of the M13 phage, plasmid DNA of HA gene of H9N2 AIV and HA-M13. M: DNA 2000 Marker, 1: M13 phage, 2: plasmid DNA of HA gene of H9N2 AIV, 3: HA-M13; (B) TEM morphology of the HA-M13. Scale bar: 200 nm; (C) Zeta potential of the HA-M13.

The HA-M13 constructs were generated through homologous recombination by incorporating the M13 phages genome and plasmid DNA containing the HA gene of H9N2 AIV. As depicted in Figure 1A, Lanes 1–3 represent PCR amplification products of M13 phages, plasmid DNA from the HA gene of H9N2 AIV, and HA-M13, respectively. Both H9N2 AIV and HA-M13 exhibited a distinct band at the 1683 bp position, which corresponded to their expected size. The HA-M13 particles were observed to be filamentous under TEM with a length of approximately 1200 nm (Figure 1B). The zeta potential of HA-M13 was determined to be −7.67 ± 1.28 mV (Figure 1C).

### 3.2. Characterize of the HA-M13/N-2-HACC/CMCS

**Figure 2.**
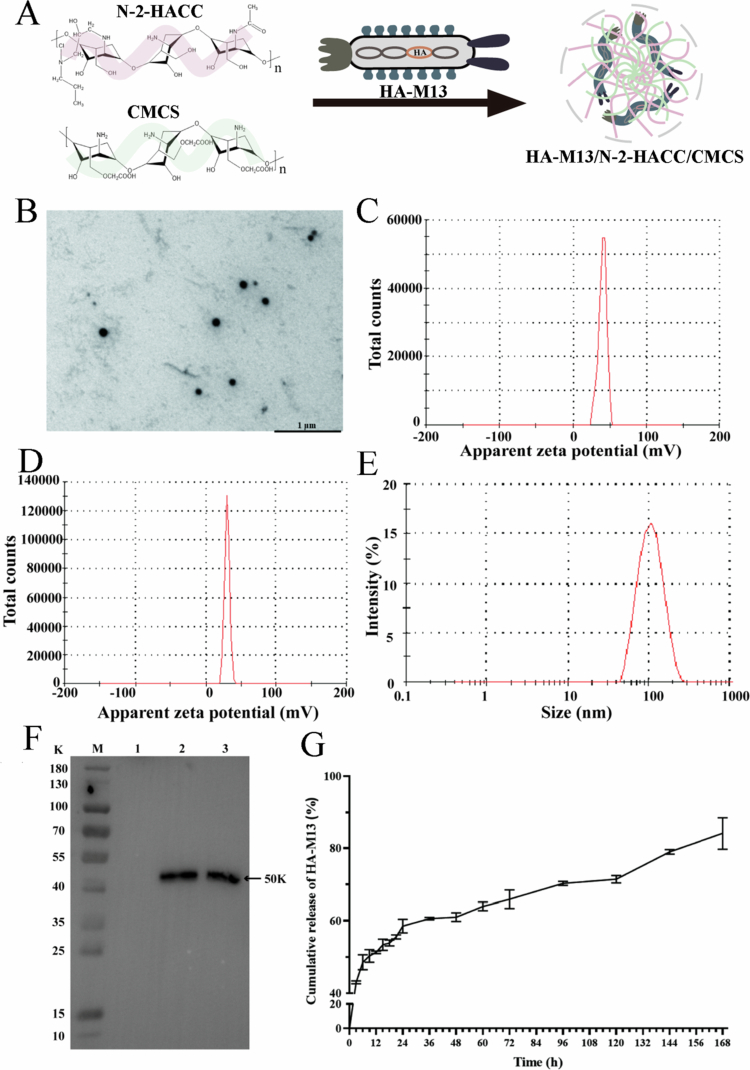
Characterization and *in vitro* release of HA-M13/N-2-HACC/CMCS. (A) The schematic diagram of the synthesis of HA-M13/N-2-HACC/CMCS; (B) TEM image of the HA-M13/N-2-HACC/CMCS, scale bar: 1 μm; (C) Zeta potential of the N-2-HACC/CMCS NPs; (D) Zeta potential of the HA-M13/N-2-HACC/CMCS; (E) Particle size and distribution of the HA-M13/N-2-HACC/CMCS; (F) Expression of HA protein by western blot. M: Protein marker (10–180 K), 1: M13 phage, 2: HA-M13/N-2-HACC/CMCS, 3: positive control; (G) *In vitro* release profiles of the HA-M13 in the HA-M13/N-2-HACC/CMCS. Data were presented as the mean ± SD (*n* = 3).

The schematic diagram of the synthesis of HA-M13/N-2-HACC/CMCS composite, shown in Figure 2A and demonstrates a uniform particle size, spherical morphology, and excellent dispersibility (Figure 2B). The zeta potential of the N-2-HACC/CMCS particles was +36.33 ± 1.37 mV (Figure 2C). The zeta potential and particle size of HA-M13/N-2-HACC/CMCS was +30.73 ± 0.57 mV (Figure 2D) and 135.24 ± 4.36 nm (Figure 2E), respectively. As HA-M13 is a little negatively charged, the zeta potential of HA-M13/N-2-HACC/CMCS composite is marginally less positively than that of N-2-HACC/CMCS. In addition, EE and LC of the HA-M13/N-2-HACC/CMCS were 74.11 ± 1.80 and 65.59 ± 7.70, respectively.

### 3.3. *In vitro* expression and *in vitro* release assay of HA-M13/N-2-HACC/CMCS

To demonstrate the successful expression of HA proteins, Western blots were carried out. As illustrated in Figure 2F, the expression of HA-M13/N-2-HACC/CMCS led to the appearance of a band of approximately 50 kDa in both the positive control groups: the HA-M13/N-2-HACC/CMCS (Lane 2) and HA gene of H9N2 AIV (Lane 3). In contrast, the M13 phage (Lane 1) did not display any bands. These results suggest that the plasmid DNA of HA gene of H9N2 AIV could be effectively encapsulated by the N-2-HACC/CMCS NPs and was expressed *in vitro*. Essentially, the N-2-HACC/CMCS NPs exhibit potential as delivery carriers for plasmid DNA. Figure 2G depicts the release profile of HA-M13 from HA-M13/N-2-HACC/CMCS in PBS buffer (pH = 7.2). The release process demonstrated an initial burst release followed by a sustained and gradual release. During the initial 9 h, the HA-M13 was rapidly released from the HA-M13/N-2-HACC/CMCS, with a cumulative release rate of 50.29% ± 1.73%. Subsequently, a gradual and sustained release occurred, surpassing 80% cumulative release at 168 h. *In vitro* release demonstrates that the N-2-HACC/CMCS NPs can act as a delivery carrier for the sustained and slow release of loaded drugs. Moreover, the HA-M13/N-2-HACC/CMCS can rapidly release a certain amount of vaccine antigen to stimulate the immune responses.

### 3.4. Stability assay of the HA-M13/N-2-HACC/CMCS

The presence of functional groups, such as -OH and -NH_2_, in chitosan confers an enhanced stability to the NPs. The outer layer of chitosan provides protection to the inner HA-M13. As shown in Figure S2A, the morphology and particle size of HA-M13/N-2-HACC/CMCS remained unchanged when stored at 4, −20, and 25 °C for 7, 14, and 30 days, respectively. The transmittance differences among each group were not statistically significant (*P* > 0.05). In Figure S2B, HA-M13/N-2-HACC/CMCS was exposed to varying pH conditions (pH 5.6, pH 7.0, and pH 10) for 14 days. No significant changes in the TEM images were observed at pH 5.6 and pH 7.0. However, at pH 10, aggregation occurred among the particles, leading to a significant difference in transmittance compared to pH 5.6 and pH 7.0 (*P* < 0.001). The decrease in zeta potential and positive charge on the N-2-HACC with increasing pH may explain this phenomenon. The reduced electrostatic repulsion between particles results in aggregation. Consequently, the HA-M13/N-2-HACC/CMCS exhibits good stability under accelerated stability testing at 37 °C (Figure S2C). Furthermore, it also demonstrates good stability under the room temperature, neutral, and weak acidic conditions.

### 3.5. *In vitro* and *in vivo* safety assay of the HA-M13/N-2-HACC/CMCS

**Figure 3.**
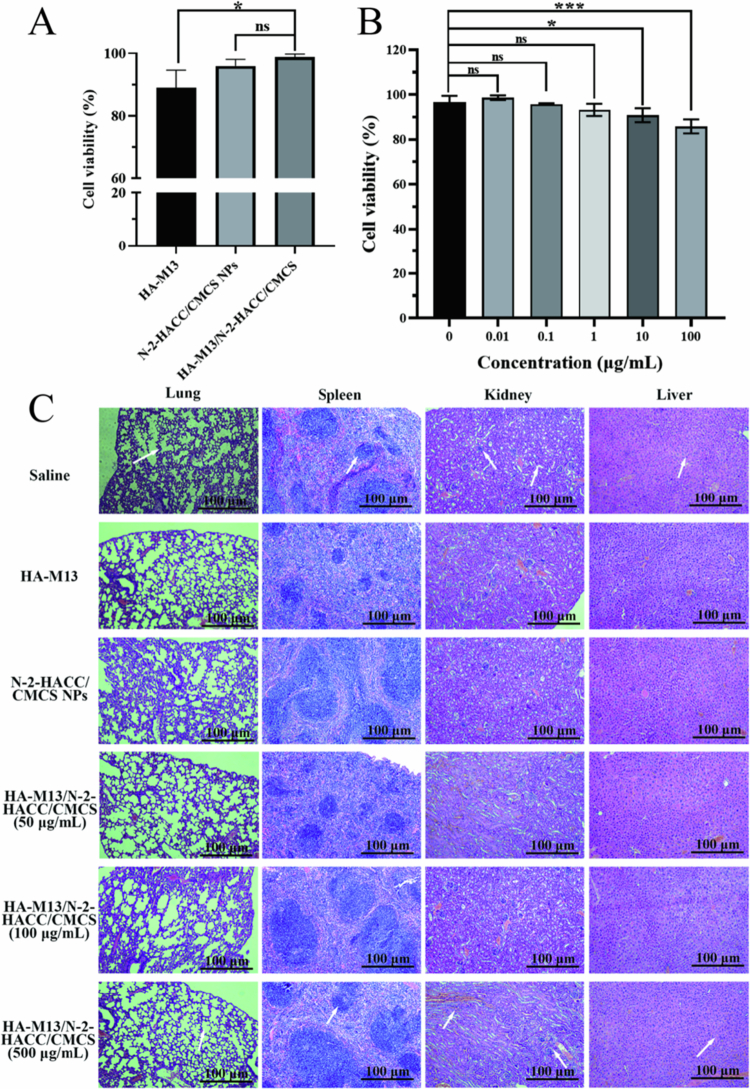
*In vitro* and *in vivo* safety assay of the HA-M13/N-2-HACC/CMCS. (A) effects of the HA-M13, N-2-HACC/CMCS NPs and HA-M13/N-2-HACC/CMCS on DC2.4 viability; (B) effects of the different concentrations HA-M13/N-2-HACC/CMCS on DC2.4 viability; (C) pathological sections of mouse organs.

The survival rates of DC2.4 cells treated with HA-M13, N-2-HACC/CMCS NPs, and HA-M13/N-2-HACC/CMCS were found to be 88.98% ± 5.45%, 95.90% ± 2.47%, and 98.76% ± 1.18%, respectively (Figure 3A). Among them, HA-M13 demonstrated the most significant impact on the viability of DC2.4 cells. The difference in cell viability between HA-M13 and HA-M13/N-2-HACC/CMCS was highly significant (*P* < 0.01). However, there was no significant difference in cell viability between the N-2-HACC/CMCS NPs and HA-M13/N-2-HACC/CMCS (*P* > 0.05), indicating that HA-M13 has inherent cytotoxicity. Nevertheless, when encapsulated by the N-2-HACC/CMCS NPs, the toxic effect of HA-M13 on cell survival was significantly mitigated. Even at a concentration of HA-M13/N-2-HACC/CMC 100 μg/mL, the survival rate of DC2.4 cells remained high at 85.85% ± 3.57% (Figure 3B). In the *in vivo* pathological section analysis, the color and texture of the tissues and organs of the mice in each experimental group were consistent with those of the control group, and no noticeable pathological changes were observed (Figure 3C). Additionally, no significant body weight changes were observed among mice after administering different materials (Figure S3). These results indicate that the HA-M13/N-2-HACC/CMCS exhibits lower toxicity to DC2.4 cells and demonstrates good biological safety.

### 3.6. Induction of DC2.4 maturation and detection of cellular uptake efficiency

**Figure 4.**
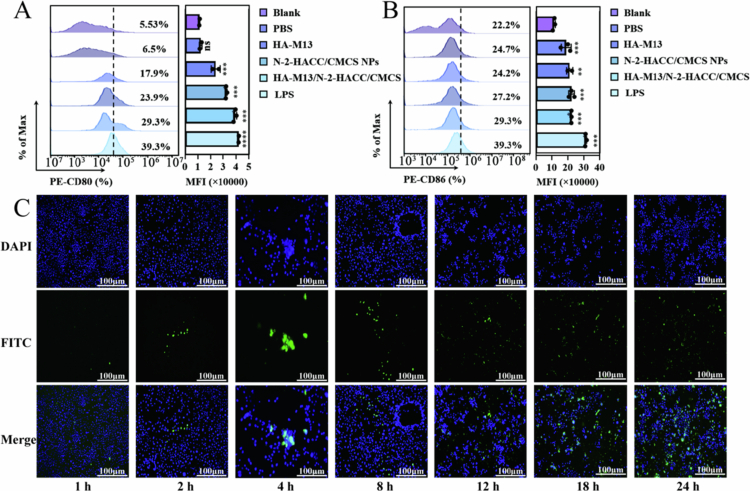
Maturation of DC2.4 and cellular uptake induced by the HA-M13/N-2-HACC/CMCS. (A) CD80 maturation of DC2.4 detected by flow cytometry; (B) CD86 maturation of DC2.4 detected by flow cytometry; (C) Uptake of the FITC-labeled HA-M13/N-2-HACC/CMCS by DC2.4 cells at the different times.

DC2.4 cells play a crucial role in antigen presentation during the immune response, delivering antigens to specific T lymphocytes and lymph nodes. They also upregulate signature markers such as costimulatory molecules (CD80 and CD86), MHC I, MHC II, and cytokines. In this study, PE-labeled CD80 and CD86 molecules were employed to assess the maturation of DC2.4. Figure 4A,B demonstrate that the difference between the HA-M13 and blank group is highly significant (*P* < 0.001). The N-2-HACC/CMCS NPs (23.9% for CD80 and 27.2% for CD86) and HA-M13/N-2-HACC/CMCS (29.3% for CD80 and CD86) significantly enhance the maturation of DC2.4 cells, with the difference compared to the blank group (5.53% for CD80 and 22.2% for CD86) being extremely significant (*P* < 0.0001). These findings indicate that N-2-HACC/CMCS and HA-M13/N-2-HACC/CMCS can promote the maturation of antigen-presenting cells, increase antigen presentation efficiency, and elicit a stronger immune response.

The cellular uptake properties of antigen-loaded NPs by APCs are crucial for the efficacy of nanovaccines. Therefore, the uptake rate of FITC-HA-M13/N-2-HACC/CMCS by DC2.4 cells was analyzed using fluorescence microscopy. Upon co-culturing FITC-HA-M13/N-2-HACC/CMCS with DC2.4 cells for 1 h, the fluorescence signal of FITC increased over time, indicating a gradual uptake of HA-M13/N-2-HACC/CMCS by DC2.4 cells between 1 and 24 h. After 24 h of culture, the FITC-labeled HA-M13/N-2-HACC/CMCS was almost entirely absorbed by DC2.4 cells (Figure 4C). This suggests that antigen-presenting cells can take up the HA-M13/N-2-HACC/CMCS after *in vivo* inoculation, promoting antigen presentation and enhancing immunity.

### 3.7. Immunogenicity evaluation

**Figure 5.**
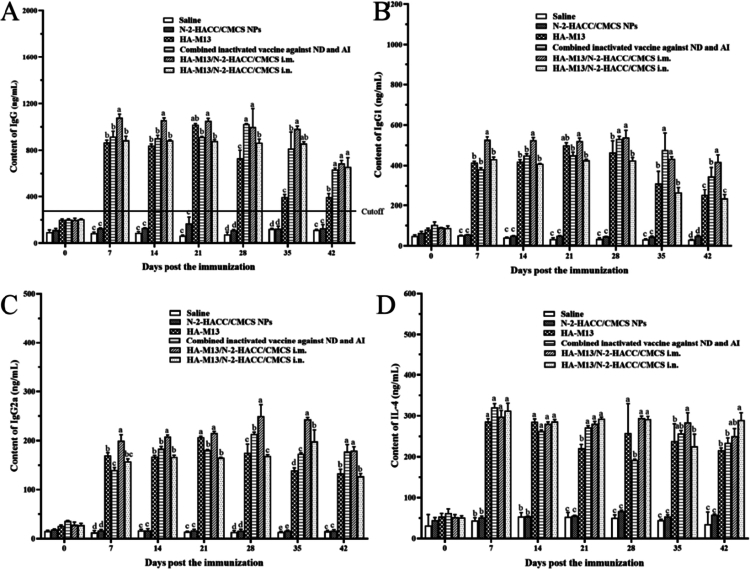
The evaluation of humoral immune efficacy of BALB/cmice immunized with the PBS i.m., N-2-HACC/CMCS NPs i.m., HA-M13 i.m., commercially combined inactivated vaccine against ND and AI i.m., HA-M13/N-2-HACC/CMCS i.m., and HA-M13/N-2-HACC/CMCS i.n. (A) The content of IgG in serum; (B) The content of IgG1 in serum; (C) The content of IgG2a in serum; (D) The content of IL-4. a–e represent data within a column without the same scripts differ significantly (*P* < 0.05).

To assess the magnitude of humoral immune response, antibody and cytokine levels in peripheral blood serum were measured. The results show that IgG, IgG1, IgG2a, and IL-4 exhibited persistently high levels throughout the 42-day period, indicating that the nanovaccine elicits long-term humoral immunity (Figure 5A–D). Compared to the control groups treated with saline and the combined inactivated vaccine against ND and AI, mice immunized with the HA-M13/N-2-HACC/CMCS i.m. exhibited a significantly augmented immune response at 7 days postimmunization (*P* < 0.05). The levels of IgG, IgG1, and IgG2a peaked at 21 and 28 days, respectively, while IL-4 reached its peak at day 7, maintaining high levels (311.05 pg/mL) for the entire 42-day period.

**Figure 6.**
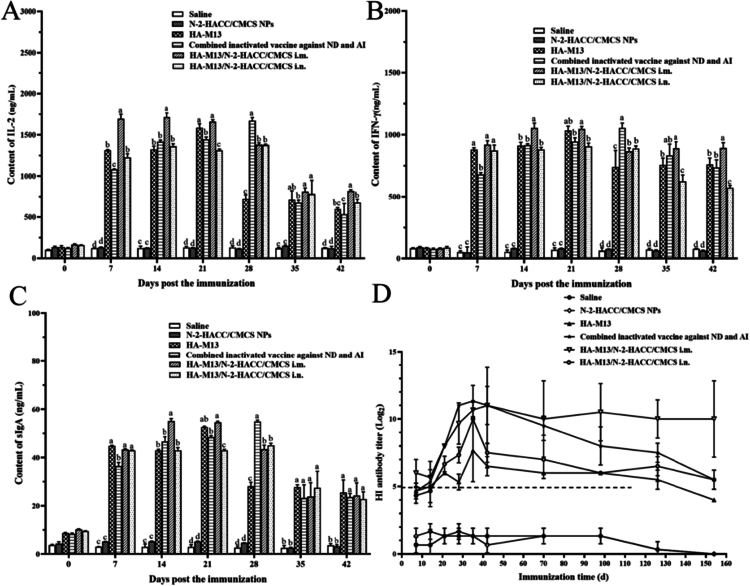
The evaluation of cellular and mucosal immune efficacy of BALB/cmice immunized with the PBS i.m., N-2-HACC/CMCS NPs i.m., HA-M13 i.m., combined inactivated vaccine against ND and AI i.m., HA-M13/N-2-HACC/CMCS i.m., and HA-M13/N-2-HACC/CMCS i.n. (A) The content of IL-2 in serum; (B) The content of IFN-*γ* in serum; (C) The content of sIgA in feces; (D) Immune duration determination. Different letters (a–d) represent a significant difference between groups (*P* < 0.05).

The application of chitosan as a delivery vehicle material holds promise for to enhancing immune responses, particularly in combating viral infections and improving cellular immunity (Bao et al. 2019). IFN-*γ* and IL-2 are typical markers of cellular immune response and play a crucial role in countering viral infections. At the cellular level, the HA-M13/N-2-HACC/CMCS i.m. group reached its zenith on the 14th day (IL-2: 1750.4 ng/mL, IFN-γ: 1069.84 ng/L), which exceeded the peak values observed in the commercially combined inactivated vaccine against ND and AI group at 28 days (IL-2: 1715.72 ng/mL, IFN-γ: 1099.53 ng/L) (Figure 6A,B). Consequently, the HA-M13/N-2-HACC/CMCS elicits more rapid and potent cellular immune responses compared to the commercial inactivated vaccine, thereby bolstering the body's resistance against viral infections.

The mucosal immune response serves as the first line of defense against invading pathogens. Consequently, mucosal vaccination is an effective approach for preventing infectious diseases. Chitosan, with its excellent mucoadhesive and stability properties, can elicit a robust mucosal immune response. The results revealed that the sIgA levels in the HA-M13/N-2-HACC/CMCS i.m. group reached a peak value of 54.74 ng/mL on the 14th day, while the commercially combined inactivated vaccine against the ND and AI group achieved a peak value of 55.53 ng/mL on the 28th day (Figure 6C). These findings indicate that the HA-M13/N-2-HACC/CMCS instigates a swifter mucosal immune response compared to the commercially inactivated vaccine while sustaining higher immune levels for a protracted duration.

### 3.8. Immune duration determination

The duration of the immune response was assessed by measuring HA-specific antibody levels using HI assay. As shown in Figure 6D, the HA-M13/N-2-HACC/CMCS i.m. group exhibited a faster production of serum antibodies and higher HI antibody titers compared to the commercial inactivated vaccine group on the seventh day postimmunization. At the 42-day evaluation, the HA-M13/N-2-HACC/CMCS i.m. group achieved a peak HI antibody titer of 2^13^, indicating that the HA-M13/N-2-HACC/CMCS induced a stronger antibody-mediated immunity than the HA-M13 alone. Remarkably, even on the 154th day postimmunization, the HA-M13/N-2-HACC/CMCS i.m. group maintained a high HI antibody titer. In contrast, the commercially inactivated vaccine group did not provide protective effects against viral infection, suggesting that the HA-M13/N-2-HACC/CMCS elicited a durable immune response that continuously protected mice from viral infections.

### 3.9. Proliferation and differentiation of T lymphocytes in spleen

**Figure 7.**
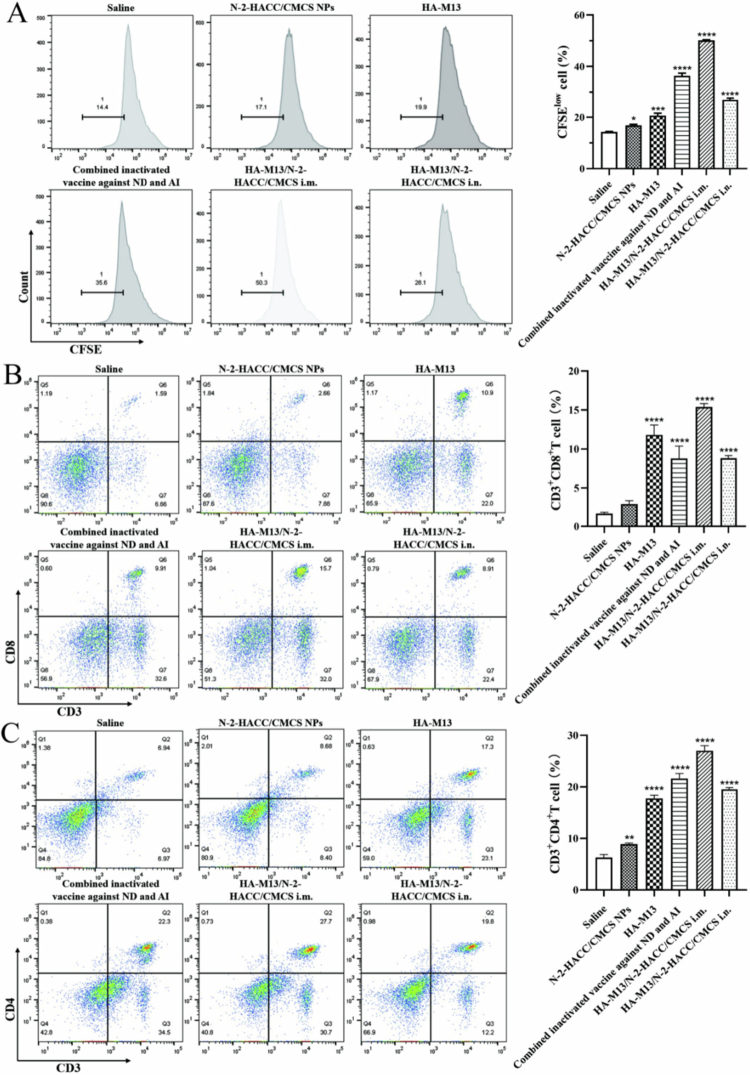
HA-M13/N-2-HACC/CMCS stimulates proliferation and differentiation of T lymphocytes in BALB/cmice. (A) Flow cytometry analysis of splenocyte proliferation; (B) The proportion of CD3^+^ CD8^+^ T lymphocytes in splenocytes was analyzed by flow cytometry; (C) The proportion of CD3^+^ CD4^+^ T lymphocytes in splenocytes was analyzed by flow cytometry.

T lymphocytes play a critical role in adaptive immune responses, and the extent of cell-mediated immune responses can be determined by assessing T lymphocyte proliferation. As depicted in Figure 7A, the HA-M13/N-2-HACC/CMCS group exhibited a significantly higher capacity to stimulate T lymphocyte proliferation compared to the group immunized with a combined inactivated vaccine against ND and AI. These results indicate that the HA-M13/N-2-HACC/CMCS can effectively elicit robust T-cell responses, exhibiting superior efficacy when compared to the combined inactivated vaccines against ND and AI. This finding is crucial for generating effective memory responses, which can provide efficient protection against H9N2 AIV infection.

T cells can be categorized into various subsets, such as cytotoxic T cells (CTLs), helper T-cells (Th), memory T cells, and more. CD8^+^ T lymphocytes serve as surface markers of CTLs, capable of directly binding to antigens presented by MHC I, thereby preventing intracellular infections. In contrast, CD4^+^ T lymphocytes, primarily surface markers of Th cells, are activated by polypeptide antigens presented by MHC II and play a role in aiding other lymphocytes and fostering immune responses (Dong et al. 2023). As illustrated in Figure 7B,C, the HA-M13/N-2-HACC/CMCS induced the highest levels of both CD4^+^ and CD8^+^ T lymphocytes compared to the other groups, indicating a particularly robust cellular immune response.

### 3.10. Protective efficacy of the HA-M13/N-2-HACC/CMCS

**Figure 8.**
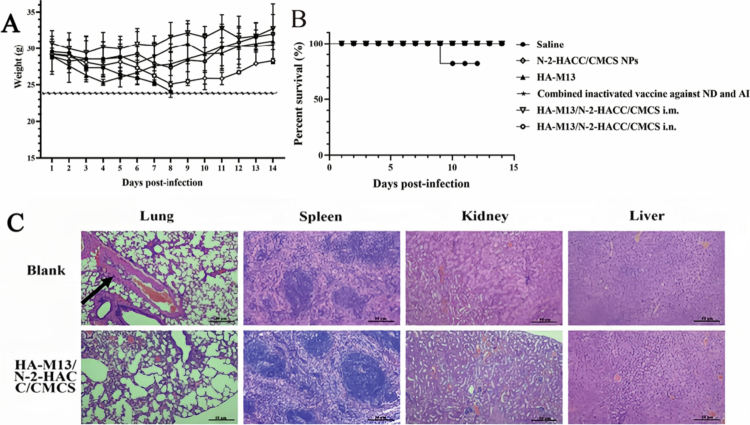
Protective efficacy of HA-M13/N-2-HACC/CMCS resistance the challenged with H9N2 AIV. (A) Body weight of BALB/cmice after challenge; (B) Survival rate of BALB/cmice in the challenge group; (C) Pathological sections of viscera of challenged mice.

Following the challenge with H9N2 AIV, the mice in the saline group experienced a significant decrease in body weight to less than 20% of their original body weight by the 11th day after the challenge (Figures 8A and S4). In contrast, the body weight of mice in the other groups decreased within 3 days, but showed an increasing trend in the subsequent days. The survival rate of mice in the saline group was 80% within 14 days. However, the survival rate of mice in the other groups was 100% (Figure 8B), indicating the protective effect of HA-M13/N-2-HACC/CMCS immunization. Pathological sections of the lungs, spleen, kidneys, and liver were observed. In the saline group, there was significant infiltration of pulmonary interstitial inflammatory cells, whereas no obvious organ lesions were observed in the HA-M13/N-2-HACC/CMCS group (Figure 8C).

On the third day after the challenge, the HA-M13/N-2-HACC/CMCS administered *via* i.m. and i.n. routes showed lower lung virus titers compared to the group treated with the commercial inactivated vaccine (Table S2). These results indicate that the HA-M13/N-2-HACC/CMCS can effectively protect mice after the challenge, exerting immune protective effects, and resisting H9N2 AIV infection.

## 4. Discussion

The primary strategies for prevention and treating AIV involve pharmacological intervention and immunoprevention. Pharmacological options encompass M2 ion channel inhibitors and neuraminidase inhibitors. However, vaccination remains the preeminent strategy for preventing and treating AIV (Carroll et al. 2016; Zhao et al. 2022). While conventional vaccines continue to evolve, their low immunogenicity, instability, and viral toxicity remain challenges to be addressed. Nanomaterials have emerged as a promising tool in vaccine development to overcome these obstacles. One advantage of bacteriophage-based vaccination is that the bacteriophage shell provides protection to the target gene after immunization, resulting in prolonged production of intracellular antigens compared to naked DNA vaccines (Malik et al. 2018). As prokaryotic viruses, bacteriophages do not replicate in eukaryotic organisms, which makes them potent immune stimulators with effective adjuvant capabilities (Dmour and Islam 2022). In this study, we combined nanomaterials and phage display technology to prepare a novel H9N2 AIV vaccine that induces humoral, cellular, and mucosal immunity, which is expected to solve the problems of short immune duration and poor immunity effect of marketed H9N2 AIV inactivated vaccines.

In this study, the M13 phage platform was employed to incorporate exogenous HA genes into the phage packaging system, improving DNA delivery stability. This design approach achieved robust cellular expression while ensuring genomic stability, highlighting its potential for DNA vaccine applications. Nanomaterials also play a crucial role in the adjuvant effect by harnessing the potential of nanoparticles and modulating particle size to enhance antigen delivery efficacy. Studies have demonstrated that particles with a size of <10 μm improve antigen recognition and delivery efficiency as they can be readily absorbed by macrophages and DCs (Khalaj-Hedayati et al. 2020; Dong et al. 2023). Chitosan, in particular, has been identified as an adjuvant with stable drug delivery properties (Mehrabi et al. 2018; Wang et al. 2021; Azizi et al. 2021). In this study, the vaccine prepared using the N-2-HACC-encapsulated M13 phages demonstrated enhanced uptake efficiency by DC2.4 cells. The surface markers CD80 and CD86 on DC2.4 cells promoted their maturation and induced immune responses more rapidly compared to commercial vaccines.

Polymeric nanomaterials, such as chitosan, exhibit excellent biocompatibility and biodegradability, making them suitable for vaccine delivery applications (Carroll et al. 2016; Xu et al. 2023). Chitosan can effectively encapsulated DNA or drugs, thereby preventing activity loss and reducing immunogenicity of biological molecules. N-2-HACC, a chitosan derivative, has been previously shown to possess outstanding sustained-release and controlled-release properties (Wang et al. 2021). The nanovaccine described in this study exhibited a biphasic release mode (Sun et al. 2016; Dudhipala and Veerabrahma 2016). The initial burst release is primarily attributed to HA-M13 phages that are loosely bound to or located near the surface of the N-2-HACC/CMCS nanoparticles. Subsequently, the slower release phase is driven by the gradual diffusion and matrix degradation of the HA-M13 phages encapsulated within the inner core of the N-2-HACC/CMCS structure.

DCs play a crucial role in antigen delivery and immune induction by capturing exogenous antigen proteins, presenting antigens through MHC I, and activating both humoral and cellular immunity (Dong et al. 2023). Chitosan accelerates DC activation and maturation through the cytoplasmic DNA sensor cGAS and STING pathways (Mehrabi et al. 2018). In our previous study, the N-2-HACC/CMCS NPs were also shown to activate macrophages and DCs and to enhance cellular immune responses through upregulation of the cGAS-STING signaling pathway, leading to increased expression of type I interferons and pro-inflammatory cytokines (Zhao et al. 2023). Taken together the reports, it is reasonable to speculate that the adjuvant effect of N-2-HACC/CMCS in the HA-M13 nanovaccine may be partially mediated through innate immune pathways such as the cGAS-STING signaling pathway. Therefore, owing to its inherent advantages, HA-M13/N-2-HACC/CMCS triggered higher levels of IL-4 and IFN-*γ* than commercial vaccines did, indicating its potential to induce more effective humoral and cellular immune responses.

Furthermore, chitosan possesses mucoadhesive properties and has been incorporated into various mucosal vaccine delivery systems to enhance immunogenicity and stability (Moresco et al. 2018; Xu et al. 2023). The principal advantage of i.n. administration lies in its ability to induce mucosal immunity at the portal of viral entry, as well as its noninvasive nature, rather than its ability to consistently generate higher systemic antibody titers (Skwarczynski and Toth 2020). Previous studies have also shown that i.n. immunization can elicit systemic humoral immune responses comparable to those induced by i.m. administration (Bolton et al. 2012). In line with these reports, our results demonstrated that both i.n. and i.m. delivery of the chitosan-based nanovaccine elicited significantly stronger immune responses and protective effects compared with the control group, while systemic antibody titers remained comparable between the two routes. These findings indicate that intranasal delivery of the chitosan nanovaccine represents an effective immunization strategy, even though it does not necessarily result in higher systemic antibody levels than i.m. administration. Lymphocyte proliferation and differentiation are crucial indicators of immunity and play significant roles in both humoral and cellular immunity (Moresco et al. 2018). Additionally, CFSE staining illustrated that the HA-M13/N-2-HACC/CMCS significantly augmented cell proliferation and immune response activation. The analysis of HA-M13/N-2-HACC/CMCS through fluorescent staining with anti-CD3, CD4, and CD8 antibodies revealed significantly higher humoral and cellular immune responses compared to commercial vaccines.

Compared with the commercial H9N2 inactivated vaccine, HA-M13/N-2-HACC/CMCS demonstrated a longer duration of protective immunity. In addition to prolonged protection, HA-M13/N-2-HACC/CMCS induced stronger cellular and mucosal immune responses, which are critical for controlling respiratory viral infections (Li et al. 2025). These results suggest potential practical advantages of the HA-M13/N-2-HACC/CMCS platform, particularly for improving immune durability and mucosal immunity. Intranasal delivery represents an attractive route for AIV vaccination due to its ability to induce local mucosal immunity and reduce reliance on injectable administration (Lian et al. 2024). Nevertheless, several challenges remain for intranasal vaccines, including rapid mucociliary clearance, antigen dilution, and the need for precise dose optimization (Bai et al. 2024). The mucoadhesive properties and sustained-release behavior of N-2-HACC/CMCS NPs may help mitigate these limitations. However, one has to recognize that a low-pathogenic H9N2 strain was used for challenge, which may limit the stringency of protective efficacy evaluation. Future studies should explore the broader applicability of this platform, including the development of vaccines targeting different AIV subtypes. In addition, evaluation in target animal models, such as poultry, will be essential to validate immunogenicity, protective efficacy, and practical feasibility under field-relevant conditions, thereby facilitating translational advancement of this nanovaccine strategy.

## 5. Conclusions

In this study, we developed a delivery platform using chitosan-encapsulated bacteriophages, constructing M13 phage-loaded quaternary ammonium chitosan NPs (N-2-HACC/CMCS) equipped with DNA vaccines. This vaccine, with high biological safety, mucosal adhesion, and stability, can be efficiently internalized by APCs, promoting their maturation and subsequent activation of T lymphocyte proliferation. Ultimately, the body produces antibodies to against viral invasion. The chitosan derivative-encapsulated phages have great potential as vaccine adjuvants and mucosal immune delivery systems and present new possibilities for vaccine development.

## Supplementary Material

Supplementary material ARRIVE_Guidelines_Checklist.pdf

Supplementary material Supplementary information.docx

## Data Availability

Data will be made available on request.
