# Supplementary material for: Genetically engineered M13 phage-mediated H9N2 DNA vaccine with enhanced mucosal and systemic immune responses in mice
Source: Drug Deliv. 2026 Feb 11;33(1):2629037. doi: 10.1080/10717544.2026.2629037 (PMC12895868; doi:10.1080/10717544.2026.2629037)
Supplement: Supplementary material — Supplementary information.docx [file IDRD_A_2629037_SM3378.docx]

**Genetically engineered M13 phage-mediated H9N2 DNA vaccine with enhanced mucosal and systemic immune responses in mice**

*Xiaohua Wang ^a. †^, Zhi Zhao ^a, †^, Mingze Shi ^a^, Shangen Xu ^a^, Xin Zhou ^b, *^, Kai Zhao ^a, *^*

*^a^ Zhejiang Key Laboratory for Restoration of Damaged Coastal Ecosystems, Zhejiang International Science and Technology Cooperation Base for Biomass Resources Development and Utilization, Taizhou Key Laboratory of Biomedicine and Advanced Dosage Forms, School of Life Sciences, Taizhou University, Zhejiang Taizhou 318000, China*

*^b^ College of Veterinary Medicine, Institute of Comparative Medicine, Yangzhou University, Yangzhou Jiangsu 225009, China*

^†^ These authors contributed equally to this study.

** Correspondence: Kai Zhao, E-mail: zybin395@126.com; Xin Zhou, E-mail: zhou_xin@126.com*


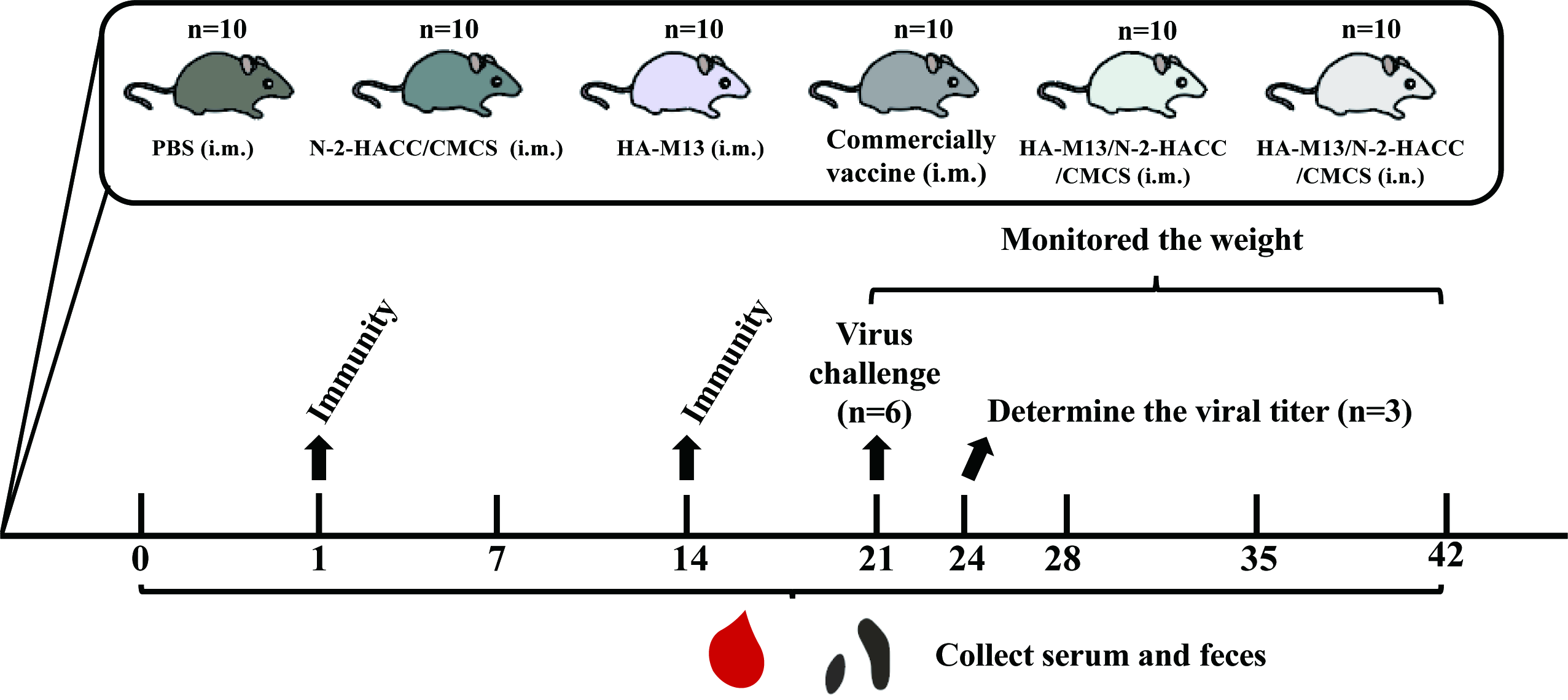


**Fig. S1** The timeline of immunization and challenge


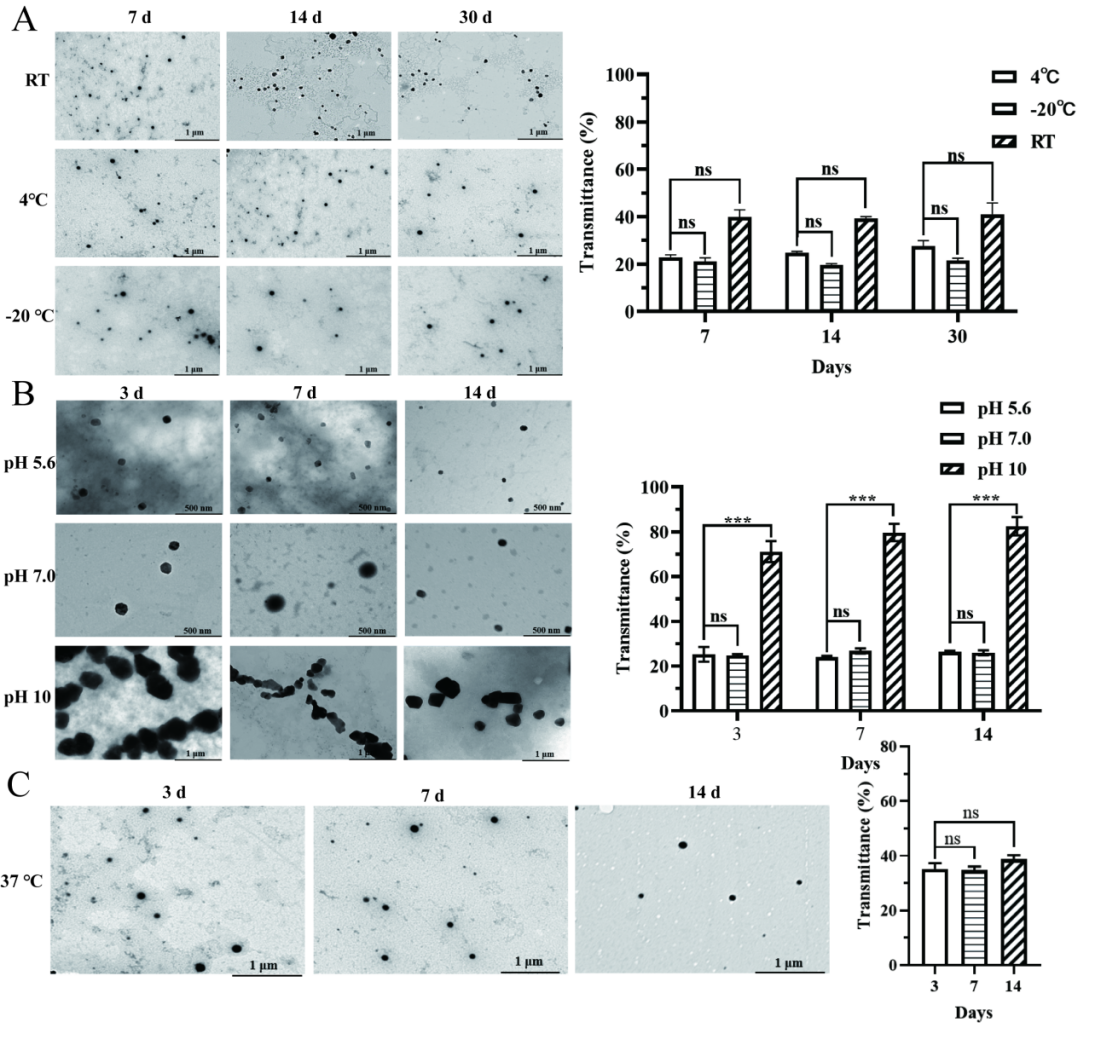


**Fig. S2.** The stability of HA-M13/N-2-HACC/CMCS under the different temperature and pH conditions. (A) TEM images of the HA-M13/N-2-HACC/CMCS at 4 ℃, -20 ℃, and 25 ℃; Transmittance of the HA-M13/N-2-HACC/CMCS at 4 ℃, -20 ℃, and 25 ℃; (B) TEM images of the HA-M13/N-2-HACC/CMCS at pH 5.6, pH 7.0, and pH 10.0; Transmittance of the HA-M13/N-2-HACC/CMCS at pH 5.6, pH 7.0, and pH 10.0; (C) TEM images of the HA-M13/N-2-HACC/CMCS at 37 ℃; Transmittance of the HA-M13/N-2-HACC/CMCS at 37 ℃.

**
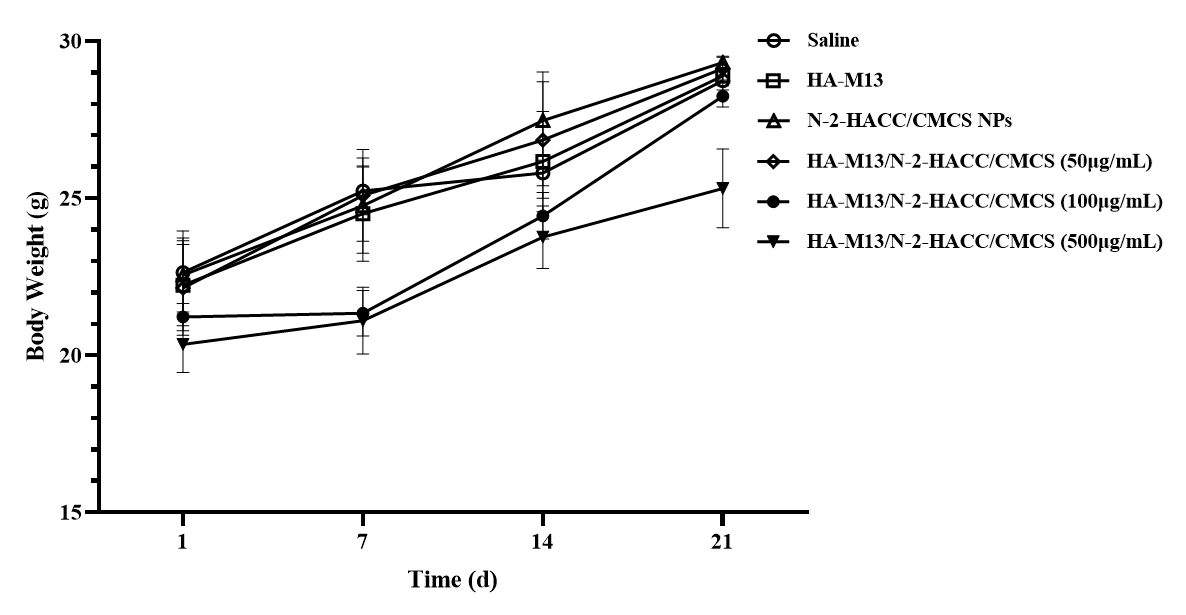
**

**Fig. S3.** Body weight change of mice after administration

**
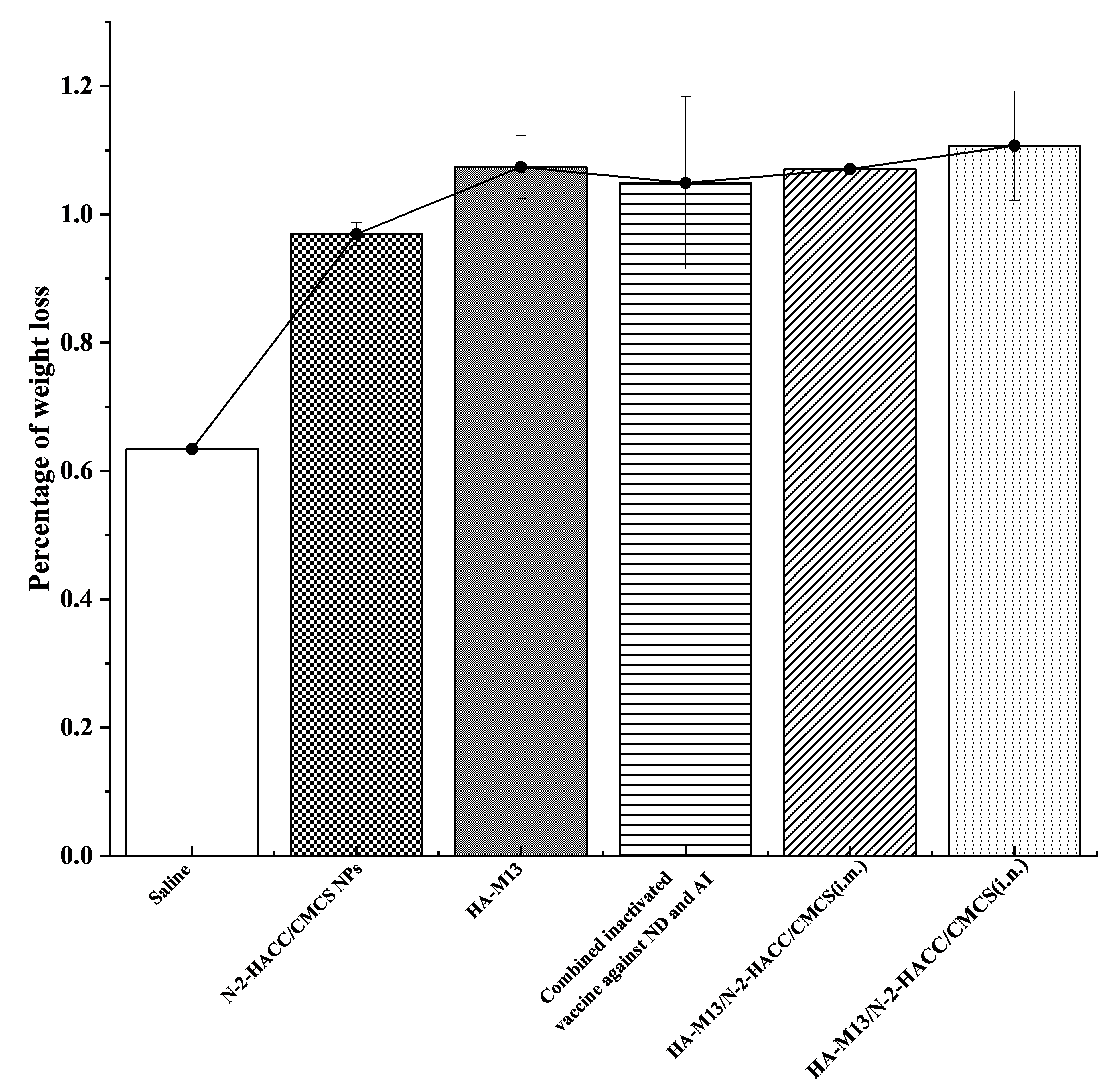
**

**Figure S4.** Percentage change in body weight of mice following H9N2 AIV challenge. Body weight was monitored and expressed as a percentage of the initial weight on day 0. Data are presented as mean±SD (n=5 per group).

**Table S1** Primer sequence of the HA and M13 phage gene

| **Primers** | **Sequence** | **Fragment** |
| --- | --- | --- |
| HA-F | ACTAGTACTGGATCCACTATGGAGACAGTATCACTA | HA |
| HA-R | TTACTTGTCGACTATACGCGTTTATATACAAATGTTGCA |  |
| M13-F | ACGCGTATAGTCGACAAGTAAAAC | M13 |
| M13-R | AGTGGATCCAGTACTAGTGTACTT |  |

**Table S2** Mean viral titers in lungs of immunized mice 3 d after challenged with the H9N2 AIV

| **Immune grouping** | **Virus titer in lungs（Log_10_ TCID_50_/mL）** |
| --- | --- |
| Saline | -6.98±0.39 |
| N-2-HACC/CMCS NPs | -5.45±0.64 |
| HA-M13 | -3.40±0.14 |
| Combined inactivated vaccine against ND and AI | -3.20±0.42 |
| HA-M13/N-2-HACC/CMCS i.m. | -2.62±0.18 |
| HA-M13/N-2-HACC/CMCS i.n. | -3.15±0.21 |
